# Unidentified chronic pelvic pain due to hematometra non-communicating left horn uterus unicornuate with history of abdominal pregnancy

**DOI:** 10.1016/j.ijscr.2024.109435

**Published:** 2024-02-28

**Authors:** Arinil Haque, Jimmy Yanuar Annas

**Affiliations:** a Residence, Department of Obstetric and gynecologic, Faculty of Medicine, Airlangga University, Dr. Soetomo Hospital Surabaya, Indonesia; b Fertility Divison Staff, Obstetric and gynecologic Airlangga, Indonesia

**Keywords:** Unicornuate uterus with non-communicating horns, Endometrioma, Abdominal pregnancy

## Abstract

**Introduction:**

This study aimed to characterize unicornuate uterus with noncommunicating horns, an uncommon Müllerian abnormality. With a 0.06 % incidence rate, this disorder can lead to endometriosis linked to retrograde menstruation or hematometra, which can cause significant pelvic pain.

**Case presentation:**

A 39-year-old woman with chief complaints of severe dysmenorrhea for five years. Despite receiving hormone therapy, the patient's symptoms persisted. She has only one living child born at laparotomy for an abdominal pregnancy 19 years ago. Upon ultrasound inspection, a 2.8 × 3 cm endometrioma was the only finding. Prior to her laparoscopic procedure, the woman had a unicornuate uterus on her right side with a normal cervix, and also a non-communicating hemiuterus in her left horn that had burst due to adhesion separation and was leaking chocolate fluid. On the left side, there was also a 3 × 3 cm endometrioma. Following that, a laparoscopic hysterectomy was carried out.

**Discussion:**

Although misread occasionally, the correct diagnosis of a unicornuate uterus with a noncommunicating horn is clinically important. The history of this patient's abdominal pregnancy may have developed in the rudimentary horn after sperm or fertilized eggs moved trans peritoneally, with life-threatening consequences if ruptured. This patient developed severe dysmenorrhea after receiving hormonal therapy, possibly caused by a noncommunicating left horn uterine hematometra and endometrioma. In this case, a laparoscopic hysterectomy was afterward chosen due to the patient's request according to her symptoms.

**Conclusion:**

Unicornuate uterine with non-communicating horns is scarce however may cause severe complications. Considered a treatment to prevent related morbidity, laparoscopy is necessary to affirm the diagnosis.

## Highlights

• This research is aim to characterize unicornuate uterus with noncommunicating horns, an uncommon Müllerian abnormality that can have major consequences so it is necessary to confirm the diagnosis, ideally prior to surgery
• This research is using the case presentation of a 39-year-old woman with chief complaints of severe dysmenorrhea for five years and a 19-year-old single child has a past of experiencing an abdominal pregnancy

## Graphical abstract

**Figure:**
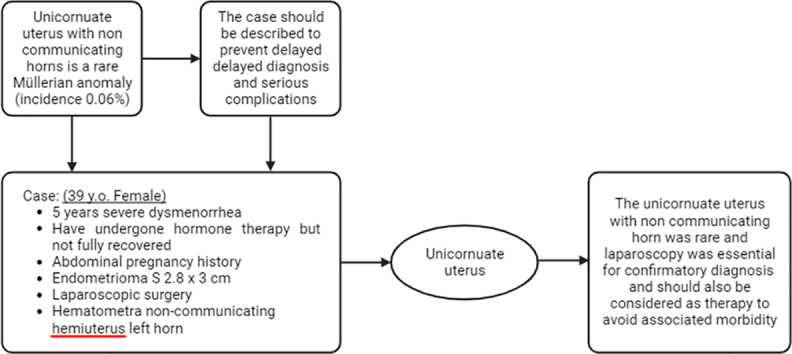

## 1 Introduction

**Fig. 1:**
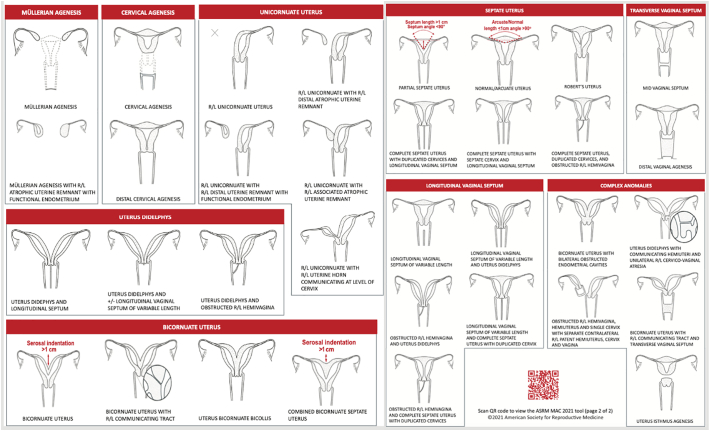
The ASRM Mullerian Anomalies Classification 2021 (MAC2021) (Adapted from Pfeifer, et al.)

The incidence of a unicornuate uterus with non-communicating horns is 0.06 %, making it a rare Müllerian abnormality. This might develop if the Müllerian ducts fail to grow completely or partially between 7 and 8 weeks of gestation [1]. In 2021, The ASRM (American Society for Reproductive Medicine) has classified Müllerian anomalies into a new category, MAC 2021, (the ASRM Müllerian Anomalies Classification 2021). Anomalies are divided into nine categories based on how they manifest themselves (Fig. 1) [2]. Regardless of whether the horn is attached or not, treatment (laparoscopic excision) is usually advised for issues like hematometra or ectopic pregnancy when there is a functional cavity in the rudimentary horn [3].

We report a case of unicornuate uterus with hematometra non-communicating left horn diagnosed during laparoscopy and never diagnosed before.

## 2 Case report

A 39-year-old woman was sent from a satellite hospital to our fertility and endocrinology outpatient clinic with chief complaints of severe dysmenorrhea. She has only one living child (a 19-year-old) born at laparotomy for an abdominal pregnancy in Cepu Hospital, 2001. She had a history of Renal stone operation in 2012. The patient felt dysmenorrhea and dyspareunia with VAS 7–8. Hormonal therapy has been given but the pain have not diminished. From the ultrasound result, there was a hypoechoic mass 2.8 × 3 cm at the left adnexa with internal echo within. The patient was diagnosed with left endometrioma and planned for laparoscopy hysterectomy and bilateral salpingo-oophorectomy due to her chief complaint.

**Fig. 2:**
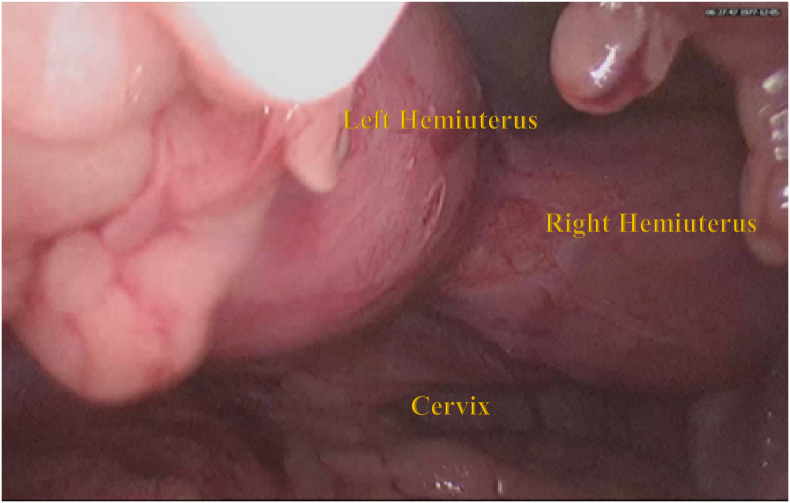
Two fundal uterus, with one cervix that connected to the right hemiuterus.

**Fig. 3:**
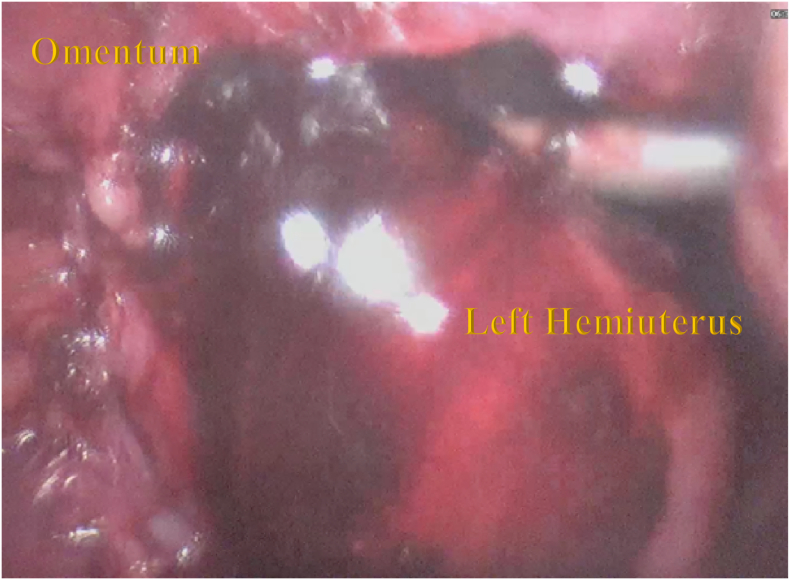
When done adhesiolysis, haematometra left hemiuterus broke, and chocolate fluid leaked.

**Fig. 4:**
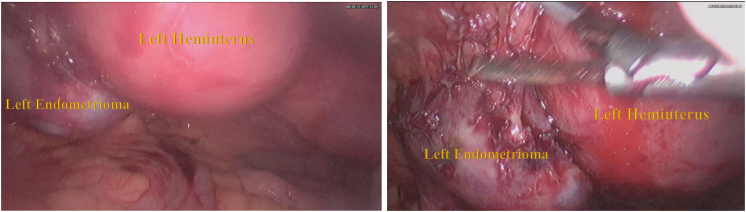
Left non-communicating hemiuterus which slightly enlarged, adhesion with endometrioma S.

**Fig. 5:**
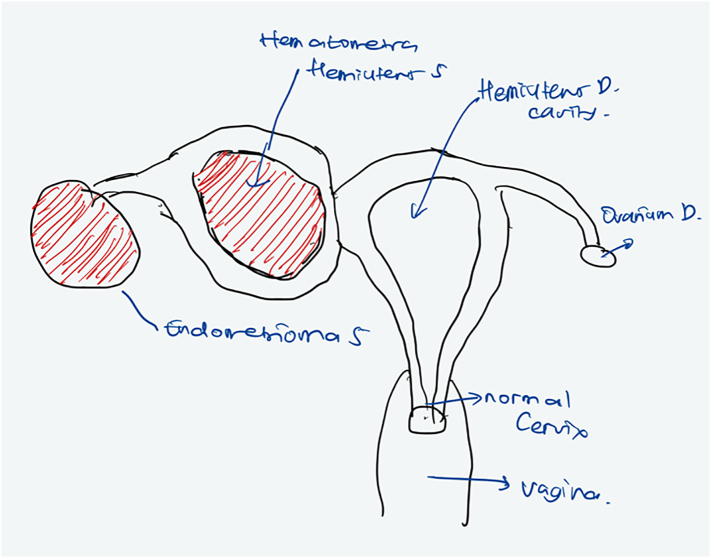
Non-Communicating Left Horn uterus unicornuate sketch.

**Fig. 6:**
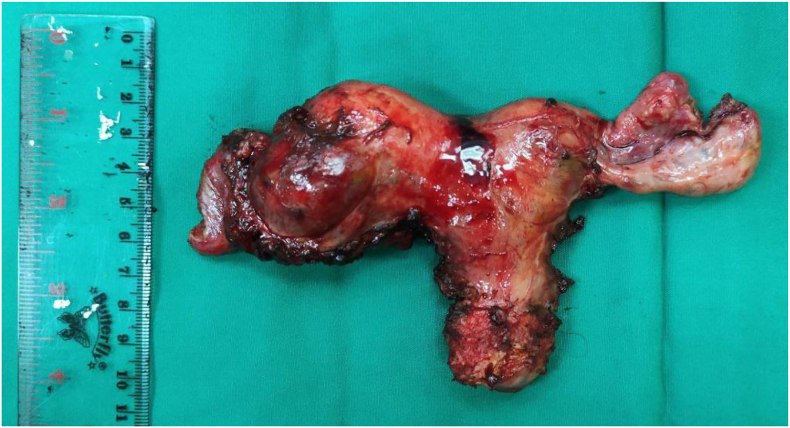
Uterus and bilateral adnexa after hysterectomy and bilateral salpingo-oophorectomy.

During the operation, it was found that there was 2 fundal uterus adhesion with the anterior abdominal wall and omentum. A right unicornuate uterus with a normal cervix and left non-communicating hemiuterus which is slightly enlarged (Fig. 2). The left one broke upon release adhesion and chocolate fluid leakage (Fig. 3). There was adhesion from the left hemiuterus with left endometrioma size 3 × 3 cm, When adhesiolisis, the left endometrioma broke and chocolate fluid leaked. This patient then performed Laparoscopy hysterectomy and bilateral salpingo-oophorectomy due to the patient's request according to her symptoms (Fig. 4, Fig. 5, Fig. 6).

## 3 Discussion

Mullerian anomalies are rare developmental anomalies of the female reproductive tract [4]. The uterus is the most common site of Müllererian anomalies, responsible for 2–8 % of infertile cases and 5–30 % of early pregnancy loss cases in women. [5]. In embryogenesis, once they start to develop medially and caudally towards the urogenital sinus, the Müllerian (or paramesonephric) ducts can be first identified at 5–6 weeks gestation. In the same way that the caudal segment of the duct conjoins in the midlines with its contralateral facet to create a uterovaginal canal that allows the uterus, cervix, and upper vagina to develop, the cranio vertical segment and the mid-horizontal segment of each duct also change into fimbria and fallopian tubes. The two Müllerian ducts are initially made of solid tissue. Later, each duct underwent internal canalization, resulting in the production of canals that are divided by a septum. Usually, it regresses after 20 weeks. The endometrial stroma and myometrium develop from the surrounding mesenchyme, whereas the lining of the united Müllerian ducts becomes uterine endometrium [6]. The unicornuate uterus, which has a prevalence of 0.06 %, is caused by abnormal or unsuccessful müllerian duct development. From the ASRM class, the unicornuate uterus is categorized into five subclasses due to the kind of uterine remnant and rudimentary uterine horn, communicating and non-communicating. A non-communicating horn's endometrium's menstruation capability is crucial for major adverse consequences such as hematometra, ectopic pregnancy, and acute abdomen. [2,3,5]. It may also cause chronic complications like endometrioma if left untreated. Torsion can occur in a non-communicating horn and be presented as a patient's shock state [7,8].

About 1 in 76,000 pregnancies occur in a unicornuate uterus with a non-communicating horn. 83 % of these cases are associated with ectopic pregnancies. Fertilized eggs or sperm may migrate through the abdomen, resulting in non-communicating horn pregnancy, an uncommon type of pregnancy. Sperm ejaculated from the vagina enter the abdominal cavity through the unicornuate uterus, where they fertilize in the fallopian tube beside the non-communicating horn before being implanted. A different possibility is that sperm passing via the unicornuate uterus fertilizes an oocyte discharged from either the left or right ovary. The fertilized egg then passes through the abdominal cavity and implants in the primitive horn [5,9]. The prevalence is about 1/100,000 to 1/140,000 pregnancies. This patient's history of abdominal pregnancy may be due to a non-communicating horn pregnancy. 50–80 % of cases have become serious complications such as uterine rupture. Uterine rupture often causes massive hemoperitoneum, leading to fetal death and maternal compromise. To avoid this morbidity and mortality, a correct diagnosis is required [5].

Dysmenorrhea is the most common symptom of this uterine anomaly, which is often misunderstood by most women as presence common among menstruating women, which may affect the delay in diagnosis [10,11]. Typically, the patient's history is entirely normal, and the existence or lack of obstructive anomalies determines the patient's symptoms. Cyclic pain is caused by the retention of menstrual secretions inside the cavity and the reflux of menstrual blood [12]. The severity of dysmenorrhea may increase each subsequent menstruation, its onset may be delayed. Progressive dysmenorrhea and chronic pelvic pain may be suspected in women with this anomaly [13]. Secondary dysmenorrhea is usually pathological and uterine malformation should always be suspected, which requires investigation and treatment [14]. The patient's severe dysmenorrhea may be due to endometriosis or hematosalpinx as a result of retrograde menstruation brought on by the non-communicating horn, or it may be the result of blood blockages forming in the non-communicating cavities of the rudimentary horn (uterine hematometra). Even with appropriate surgical or pharmaceutical care, the symptoms may return. Surgery as a cytoreductive treatment that eradicates the disease, however endometriosis can reoccur. Even though hormonal therapy has been given to this patient, the dysmenorrhea remained incurable [6,14,15].

Several complications arising from these Müllerian anomalies demonstrate the importance of early diagnosis. It's crucial to recognize that patients with dysmenorrhea may have noncommunicating uterine horns, even though the diagnosis of this uterine deformity is still difficult. When diagnosing endometriosis, obstructive Müller anomaly should be excluded [16]. These delays may result in persistent symptoms and adversely affect quality of life [17]. Although the diagnosis is only successful in 14 % of cases, it might be suspected based on the patient's medical history, physical examination, hysterosalpingography, ultrasonography, and MRI. [18,19]. As shown in our case, the unicornuate uterus is difficult to diagnose with conventional ultrasonography [7] This patient was diagnosed with endometriosis before surgery, and uterine abnormalities were diagnosed after surgery. MRI remains the imaging modality of choice for identifying, classifying, and guiding surgical treatment of various uterine anomalies, especially for characterizing Müllerian anomalies [20]. Although intravenous pyelography (IVP) is less helpful in the presence of magnetic resonance imaging (MRI), it can still be utilized as a diagnostic technique to rule out related renal problems. Initial MRI and IVP are essential for surgical planning because they offer a more comprehensive picture of the anatomy of the pelvis and the variety of myometrial connections [7].

Using a laparoscopy, we were able to eliminate the endometrioma and the non-communicating horn to alleviate the patient's complaints and validate the suspicion of a unicornuate uterus with a non-communicating horn [17]. Reduced postoperative pain, reduced adhesion formation, and a shorter hospital stay are the benefits of laparoscopic surgery. With smaller incisions, the abdominal scarring is minimal. The preferred course of treatment after a thorough preoperative evaluation and the absence of any concurrent urinary tract disorders is laparoscopic excision of the non-communicating horn, which is a safe procedure [11]. At the time of surgery, it is suggested to use hysteroscopy to determine the normality of the unicornuate uterine cavity and to perform trans-illumination of the unicornuate uterus to confirm the anatomical planes between the horns and to minimize the risk of penetrating into the unicornuate uterine cavity when the transaction of the non-communicating Retroperitoneal dissection is recommended to reduce the risk of ureteral injury due to the high risk of ureteral malposition and the presence of abnormal vessels [21]. As soon as the non-communicating horn and its accompanying canal are diagnosed, especially in adolescents, laparoscopic excision should be carried out. This will relieve the symptoms of dysmenorrhea and stomach pain while also preventing more significant consequences like endometriosis, infertility, ectopic pregnancy, and rupture of the non-communicating horn [22]. Regarding the patient's desires and the ineffectiveness of hormone therapy in addressing her primary complaint, laparoscopic hysterectomy and bilateral salpingo-oophorectomy were performed on this patient.

## 4 Conclusion

Although uncommon, unicornuate uteri with non-communicating horns can have major consequences. It is necessary to confirm the diagnosis, ideally prior to surgery. Laparoscopy should be considered a treatment to prevent accompanying morbidity and is necessary to confirm the diagnosis.

## Scare reported

This study has been reported in accordance with the SCARE criteria [23].

## Consent

This case report has approved informed consent from the patient.

## Ethical approval

There is no ethical clearance was declared. This case report has approved informed consent from the patient.

## Funding

This research received no specific grant from any funding agency in the public, commercial, or not-for-profit sectors.

## Author contribution

AH: as a writing, and analysis of the paper.

JYA: as a consultant, and supervisor of this patient.

## Guarantor

Arinil Haque.

## Research registration number

N/A.

## Conflict of interest statement

The authors declare that they have no known competing financial interests or personal relationships that could have appeared to influence the work reported in this paper.
